# Revealing RELL1 signaling relying on the phosphoregulatory network analysis

**DOI:** 10.3389/fbinf.2026.1939325

**Published:** 2026-09-07

**Authors:** Pathiyil Sajini Sekhar, Diya Sanjeev, Amal Fahma, Suhail Subair, Athira Perunally Gopalakrishnan, Prathik Basthikoppa Shivamurthy, Rajesh Raju, Sowmya Soman

**Affiliations:** Centre for Integrative Omics Data Science (CIODS), Yenepoya (Deemed to Be University), Mangalore, Karnataka, India

**Keywords:** MAPK, mTOR, neurological disorders, phosphoproteomics, phosphosites, RELL1

## Abstract

**Introduction:**

RELL1 (Receptor Expressed in Lymphoid Tissues-Like 1) is a member of the TNF receptor superfamily. Although the physiological ligands and mechanistic insights into cell signaling still remain to be elucidated, its over-expression is associated with poor prognosis and enhanced immune cell infiltration in cancers, marking it a potential oncogenic driver and immunotherapeutic target. Despite extensive phosphoproteomic evidence indicating that RELL1 undergoes phosphorylation, the functional significance of its phosphosites has not been systematically investigated.

**Methods:**

Global human phosphoproteomic datasets were systematically analyzed to identify predominant RELL1 phosphosites. The two most frequently detected phosphosites were selected as predominant sites for phosphosite-centric analysis. To explore their functional significance, their highly co-phosphoregulated sites in other proteins across diverse conditions were examined. A stringent criteria was applied to determine protein phosphosites that showed either positive or negative co-regulation with predominant sites. These co-regulated sites were further analyzed within known interacting proteins, and predicted the upstream regulators of RELL1, followed by enrichment analysis.

**Results:**

Exemplified by the detection of its phosphorylated forms in 474 qualitative and 150 quantitative phosphoproteomic datasets across multiple experimental conditions, we propose phosphorylation as a potential regulatory mechanism associated with RELL1 function. However, the functional role of any of the phosphosites in RELL1 phosphosites remains unknown. Among the detected phosphosites, S244 and S161 were the most frequently observed sites perturbed across datasets, and hence, considerably the predominant sites. Co-phosphoregulation analysis revealed plausible regulatory networks comprising interacting proteins and predicted upstream kinases. Bioinformatics analyses revealed strong associations between these phosphosites and biological processes such as nuclear transport and cell cycle regulation.

**Conclusion:**

This systems-level phosphoproteomic investigation provides the potential phosphoregulatory map for RELL1 phosphosites, which could lay a foundation for further experimental investigations as well as in establishing its functional roles contributing to advancements in RELL1 signaling events relevant to immuno-oncotherapy.

## 1 Introduction

Receptor expressed in lymphoid tissues (RELT) is a type 1 transmembrane glycoprotein and member of the TNF receptor superfamily, characterized by a cysteine-rich extracellular domain and showing significant homology to other TNFR family members. It is predominantly expressed in hematopoietic tissues and has been reported to promote T Cell proliferation (Sica et al., 2001). The two RELT homologs, RELL1 (Receptor expressed in lymphoid tissues like 1) and RELL2 (Receptor expressed in lymphoid tissues like 2), interacts with RELT, together constituting RELT family (RELTfm) of proteins, a unique subfamily within the TNF receptor superfamily (Cusick et al., 2006).

The members of the RELTfm exhibit significant sequence homology within the transmembrane domain. RELT shares 32% sequence similarity with RELL1 and 27% with RELL2 and are 40% identical to each other (Cusick et al., 2006). These conserved regions in the transmembrane domain are particularly important in maintaining its structural and functional integrity. RELTfm genes have orthologs in amphibians, reptiles, birds, and fish, highlighting their strong evolutionary conservation (Cusick et al., 2023). RELL1 is expressed ubiquitously at the transcript level, with the highest levels of expression found in the testis, placenta, and various organs like heart, brain, colon, ovary, small intestine, skeletal muscle. However, RELL2 expression is more restricted to tissues like the thymus, spleen, testis, brain, and placenta (Cusick et al., 2006). The RELT gene is encoded on chromosome 11 (11q13.4) (Sica et al., 2001), while the RELL1 gene is on chromosome 4 (4p14), and the RELL2 gene is on chromosome 5 (5q31.3). The RELL1 has a molecular weight of 29,340 Da and consists of 271 amino acids while RELL2 has 32,405 Da and 303 amino acids respectively (UniProt Consortium, 2023). The RELT functions as an orphan receptor whereas the RELL1 and RELL2 do not function as receptors themselves but instead act as adaptor proteins that modulate signaling by RELT (Cusick et al., 2006). Unlike typical TNF receptors, RELL1 and RELL2 have smaller extracellular domains and lack the cysteine-rich regions required for binding with TNF ligands (Cusick et al., 2006).

RELL1 is an emerging signaling protein with functional significance in various diseases, including cancers, infectious diseases, chronic inflammatory conditions, and neurological conditions (Jin et al., 2020). Recent studies have discussed the oncogenic role of RELL1 in glioblastoma, an aggressive brain tumor with a poor prognosis, where it is overexpressed and contributes to tumor progression and modulation of the immune microenvironment. RELL1 functions as an oncogene and is associated with a poor prognosis and enhanced immune cell infiltration, marking it a potential oncogenic driver and immunotherapeutic target (Jin et al., 2020). Supporting its clinical relevance, proteome profiling of glioblastoma-associated surfaceome was done using cell surface biotinylation and shotgun proteomics, which identified RELL1 as one of the 11 GBM-specific surface proteins, some of which are also mutated. RELL1 remains non-targeted by the currently available drugs or existing therapies, marking it as a novel and unexplored candidate for Chimeric Antigen Receptor (CAR) T Cell therapy, mRNA vaccines, or other immunotherapeutic strategies (Rose et al., 2021). In tuberculosis, RELL1 is upregulated in infected macrophages by activating the mammalian target of rapamycin (mTOR) pathway and blocking autophagy, which helps *Mycobacterium tuberculosis* survive and avoid the immune system (Feng et al., 2020). In cardiovascular diseases, the circular RNA circ-RELL1 has been found to enhance endothelial inflammation by activating the Myeloid Differentiation Primary Response Gene (88) (MyD88)/Nuclear Factor Kappa B Subunit 1 (NFKB1) pathway, which plays a role in the development of atherosclerosis (Huang et al., 2020). Evidence of phenotypic characterization of RELL1 knockout mice generated by targeted deletion of exon 4 in the C57BL/6NJ background revealed significant neurological abnormalities, indicating that RELL1 plays a critical role in nervous system development and brain function (Dickinson et al., 2016; Cusick et al., 2023).

RELL1 interacts with proteins such as Filamin A (FLNA) and MyoD Family Inhibitor Domain Containing (MDFIC), which play crucial roles in cytoskeletal structure and transcription control. These interactions have an impact on the activation of p38 Mitogen-Activated Protein Kinase (MAPK) and the apoptotic pathway. The involvement of RELL1 in various signaling cascades supports its functional relevance in cell survival, migration, and cancer (Cusick et al., 2020; Cusick et al., 2021). Despite these diverse roles, the regulatory mechanisms that control the activity of RELL1 remain poorly understood. Considering its interaction with central signaling pathways like MAPK/p38, mTOR, and NFKB1, phosphoproteomic (phosphocentric) analysis is a potent and essential next step. Phosphorylation is a master switch in signaling pathways that fine-tunes protein activity, localization, and interaction. RELL1 is already known to interact with Oxidative Stress Responsive Kinase 1 (OXSR1), a kinase that phosphorylates signaling proteins involved in immune and stress responses (Cusick et al., 2006), and is possibly regulated by phosphorylation. However, due to lack of high-resolution structural studies such as X-ray crystallography or cryo-electron microscopy, it remains difficult to understand the specific conformational changes, regulatory mechanisms, and interaction domains of RELL1 that may be influenced by phosphorylation or protein-protein interactions. A systems-level approach is essential for understanding the signaling repertoire of RELL1 in diverse biological conditions.

Hence, the current study was undertaken to systematically analyse the global phosphoproteomic datasets pertaining to RELL1 to identify the RELL1 phosphosites. Additionally, we carried out a co-regulation analysis of the predominant RELL phosphosites with phosphosites on other proteins to probe into its known and predicted kinases and interactors. The data obtained from this study could serve as a knowledge base for the regulatory network as well as the functional associations relevant to RELL1.

## 2 Materials and methods

### 2.1 Comprehensive curation, screening, and analysis of global phosphoproteomic datasets with RELL1 phosphosites

An extensive literature review on PubMed using the keywords “phosphoproteomics” OR “phosphoproteome” NOT “Plant” NOT “Review” was carried out to curate global high-throughput phosphoproteomics datasets derived from human cell lines. From these studies, we compiled datasets that included the class 1 phosphosites (localization probability ≥75%; A-score >13) of RELL1. The curated datasets were obtained from the Supplementary Material of published articles and were categorized based on the type of phosphosite enrichment methods (STY, ST, or Y). The datasets were grouped into two categories: qualitative profile datasets (test conditions and control are treated as independent datasets) and quantitative differential datasets (test conditions vs. corresponding control). The curated phosphoproteomic datasets included in this study were generated across diverse experimental conditions using different mass spectrometry platforms, sample preparation workflows, phosphopeptide enrichment strategies, and labeled or label-free quantification methods, resulting in inherent technical variability. As uniformly processed raw data were not available for all studies, cross-dataset normalization and batch-effect correction could not be performed. To minimize potential bias arising from this heterogeneity, a standardized phosphoproteomic workflow routinely applied in our laboratory was employed (Priyanka et al., 2024; Sekhar et al., 2026). The phosphosites with a fold change cut-off of ≥1.3 for upregulation, ≤0.76 for downregulation, and statistically significant with p-value <0.05 (based on primary analysis in each study) were considered as differentially regulated. Individual proteins in each dataset were mapped to their corresponding gene symbols to the latest HUGO Gene Nomenclature Committee (HGNC) (downloaded on 30.05.2023) (Povey et al., 2001), and each phosphosites was mapped to the corresponding UniProt (downloaded on 13 May 2023) (UniProt Consortium, 2023) accession versions using our in-house mapping tool to ensure uniform mapping. To enhance visualization and analysis, we annotated the experimental conditions or biological contexts of each phosphoproteome dataset using a standardized format.

### 2.2 Sequence coverage profiling and peptide map analysis of RELL1

To represent and visualize the peptide sequence, post-translational modifications (PTMs) on serine (S), threonine (T), and tyrosine (Y) residues, and the overall sequence coverage of the RELL1 protein, we analyzed the total sequence coverage and its Class I phosphosites obtained from global human cellular phosphoproteomic profile data. This analysis focused on identifying the unique and frequently modified RELL1 peptide sequences, particularly those containing multiple PTMs across diverse biological contexts. The Sequence Coverage Visualizer (SCV), a protein structure prediction tool, was used to visualize sequence coverage and Class I phosphosites (Shao et al., 2023).

### 2.3 Identification of predominant phosphosites in RELL1

To determine the predominant phosphosites of RELL1 from human cellular qualitative data enriched in the serine/threonine (S/T) and tyrosine (Y) phosphoproteomes, we ranked phosphosites according to their frequency of detection across different datasets. The predominant RELL1 phosphosites were selected primarily based on the differential frequency (how frequently they were observed) in quantitative datasets to investigate the co-differentially regulated phosphorylation sites in other proteins (CPOPs). The frequency of RELL1 phosphosites in the compiled qualitative and quantitative datasets was visualized using lollipop plots generated with the M2Viz tool (Rafi et al., 2026).

### 2.4 Analysis of phosphosites of other proteins that are co-differentially regulated with RELL1 predominant sites

To analyse the phosphosites of other proteins that are positively or negatively co-differentially regulated with the predominant phosphosites of RELL1, we examined a wide range of quantitative differential phosphoproteomic datasets. These datasets were from different experimental conditions, biological systems, and methods. Due to the huge number of datasets, reanalyzing the raw data was not practical. Instead, we classified datasets based on the regulation patterns of each predominant site upregulated (U) or downregulated (D) and examined the differential expression. This classification strategy of CPOPs is based on the previous methodologies established by our lab (Sanjeev et al., 2024; Lalu et al., 2025; Mahin et al., 2025). For each predominant site, CPOPs were grouped based on their co-regulation patterns as UU (both upregulated), DD (both downregulated), UD (predominant site upregulated, CPOPs downregulated), and DU (predominant site downregulated, CPOPs upregulated). CPOPs found consistently in UU and DD conditions were considered positively co-regulated (UUDD), while those in UD and DU categories were considered negatively co-regulated (UDDU). Considering certain drawbacks, this approach offers a basis for predicting proteins that may be co-regulated with RELL1.

### 2.5 Approaches for filtering and identifying CPOPs across the datasets

The selection of highly co-regulated proteins was based on multiple stringent criteria. To assess the likelihood and confidence of co-regulation, we counted the number of datasets or conditions where either the predominant phosphosite or the CPOPs were both detected as differentially regulated. Fisher’s Exact Test (FET) was employed to assess the statistical significance of associations between specific phosphosites and experimental conditions. A one-sided FET was performed by constructing a contingency table for the corresponding RELL1 and other protein sites.

Fisher’s exact test (FET):

$$\sum P=\frac{\left(a+b\right)!\left(c+d\right)!\left(a+c\right)!\left(b+d\right)!}{n!}\sum_{i}\frac{1}{a_{i}!b_{i}!c_{i}!d_{i}!}$$

Where a (n_00) represents the number of experimental conditions in which neither the RELL1 phosphosites nor the CPOPs were detected; b (n_U0+n_D0+n_0U + n_0D) denotes the number of experimental conditions in which only one of the two phosphosites was detected (either up- or downregulated), while the other was absent; c (n_UD + n_DU) corresponds to the number of experimental conditions exhibiting negative regulation between the two phosphosites; d (n_UU + n_DD) represents the number of experimental conditions showing positive regulation.

To minimize potential biases, rigorous filtering criteria were applied. Specifically, CPOPs with a p-value <0.05 that fall into the UUDD or UDDU categories of co-regulation with the predominant phosphosites were considered, along with those CPOPs that satisfy a ratio representing 10% of the total frequency of the specific predominant phosphosite, were selectively chosen for further analysis. Additionally, proteins were required to be reported in at least three PubMed-indexed studies (PMID confidence ≥3) and detected in at least three independent experimental conditions (code confidence ≥3). Co-regulations of phosphosites from other proteins that met these filtering criteria were classified as high-confidence or highly co-regulated proteins. All subsequent analyses were conducted based on this selection.

### 2.6 Co-occurrence analysis of RELL1 predominant sites

To identify the mutual associations and co-regulation patterns of phosphosites within RELL1, a co-occurrence analysis was conducted, where we particularly identified the co-differential regulation patterns of phosphosite pairs within RELL1. Each differential data set having multiple phosphosites of RELL1 detected in the same experimental condition was extracted. For each pair, we separately calculated the UU, UD, DD, and DU frequencies and further assessed their positive co-regulation pattern using ∑ (nUU + nDD)/∑ (nUD + nDU) and negative co-regulation pattern using ∑ (nUD + nDU)/∑ (nUU + nDD). This approach allowed us to assess potential interdependence between phosphorylation sites and determine their functional proximity in co-regulation dynamics. A heatmap to visualize co-occurrence of phosphosites was generated using matplotlib and seaborn libraries in Python (Han and Kwak, 2023), where a positive or negative co-occurrence with a frequency above 3 is coloured in red or green gradients, respectively. The phosphosite that showed a higher frequency of occurrence (>3) was considered to be co-occurring or having similar functions.

### 2.7 Analysis of proteins and phosphosite-specific interactors with RELL1 phosphosites

CPOPs that exhibited either positive or negative correlations with specific phosphosites in RELL1 were selected for further analysis. The interaction landscape of RELL1 was studied by compiling the experimentally known protein-protein interactions extracted from databases such as the Human Protein Reference Database (Keshava Prasad et al., 2009), Biomolecular Interaction Network Database (Bader et al., 2003), Biological General Repository for Interaction Datasets (BioGRID) (Oughtred et al., 2021), ConsensusPathDb (version 35) (downloaded May 2023) (Oughtred et al., 2021), CORUM comprehensive resource of mammalian protein complexes (downloaded March 2023) (Tsitsiridis et al., 2023), and RegPhos (version 2.0) (downloaded May 2023) (Huang et al., 2014).

### 2.8 Identification of upstream kinases of RELL1

The upstream kinases associated with specific RELL1 phosphosites were predicted using multiple tools, including NetworKIN (downloaded on 04.01.2023) (Linding et al., 2008) and AKID (downloaded on 24.05.2023) (Parca et al., 2019), and those derived from a high-throughput approach based on an *in vitro* screen of 385 kinases as provided in the *In vitro* Kinase-to-Phosphosite database (iKiP-DB) (Mari et al., 2022) were extracted. Significantly, all the kinases of RELL1 were categorized by (Johnson et al., 2023), based on the synthetic peptide screening for the assessment of the substrate specificity of the kinome, with a cutoff of the 90th percentile, and were also extracted for analysis as described in subsequent sections. Additionally, the co-regulated network of kinases and phosphatases was determined through HGNC (Seal et al., 2023) and Co-phosphorylation-based Kinase-Substrate Interaction Prediction (CoPhosK) (Ayati et al., 2019).

### 2.9 Functional insights and disease association of RELL1 through gene enrichment analysis

The functional gene enrichment analysis of co-regulated proteins with RELL1 predominant phosphosites was performed using Enrichr (Kuleshov et al., 2016). Additionally, we performed disease enrichment analysis using Enrichr, based on the Jensen disease databases (Grissa et al., 2022). Cytoscape (Shannon et al., 2003), Path Visio 3 (Shannon et al., 2003; Kutmon et al., 2015), Rawgraph (https://www.rawgraphs.io/), and BioRender (2023) (https://www.biorender.com/) were used for the visualization of the results and pathways.

## 3 Results

### 3.1 Global phosphoproteomic dataset analysis

The function of a protein is largely regulated by PTMs, such as glycosylation, acetylation, phosphorylation, and more. These modifications are responsible for the protein’s activity, stability, localisation, and interactions. With the advancement of high-throughput technologies, phosphoproteomics research has gained much attention over the past few years. Large-scale phosphoproteome datasets were generated over years across a wide range of biological and experimental conditions, which showed considerable variation in the phosphorylation patterns identified.

To analyze the functionally significant phospho-signaling patterns associated with RELL1, we screened over 3,825 publicly available human global cellular phosphoproteomics datasets and identified 150 differential datasets and 474 profile datasets containing class-1 RELL1 phosphosites. After the comprehensive analysis and an extensive mapping of class 1 phosphosites across these datasets, eighteen distinct phosphosites were identified in profiling, and nine were found to be differentially regulated across various experimental conditions, including cancers, infections, and hormonal stimulation of cells. The profiling and differential datasets of class 1 phosphosites of RELL1 are given in Supplementary Materials 1A,B.

### 3.2 Identification of predominant phosphosites of RELL1

RELL1 phosphosites were ranked after an extensive analysis of the differential datasets based on frequency of detection in diverse experimental conditions. Among the nine differentially regulated phosphosites, S244 and S161 were identified as the predominant sites. The detection of these phosphosites, S244 and S161, across 65 and 64 different experimental conditions, respectively, highlights their consistent presence and potential biological importance. Such regular occurrence in multiple contexts suggests that these sites may play crucial roles in regulatory functions, promising further exploration of their functional significance in cellular signaling and disease mechanisms.

**FIGURE 1:**
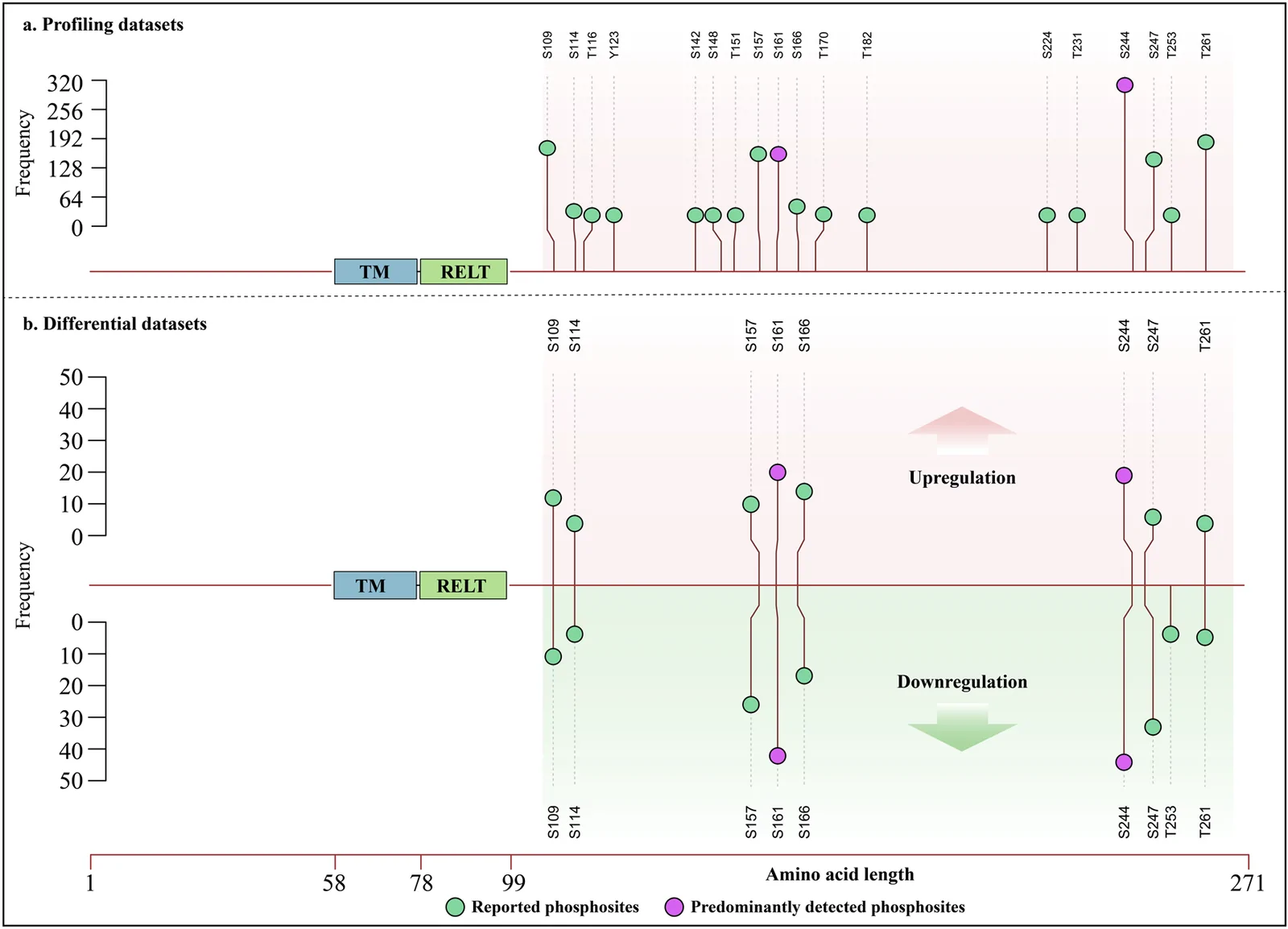
Lollipop plot visualization of phosphosites within RELL1 with their detection frequencies in global cellular phosphoproteomics datasets. **(a)** Phosphosites detected in the qualitative profiling datasets **(b)** Phosphosites detected in the quantitative differential datasets.

These predominant phosphosites were located outside the functional domain of RELL1. The frequency of all eighteen phosphosites in RELL1 qualitative profiling (Figure 1a) and nine in quantitative differential datasets (Figure 1b) is depicted in the lollipop plot.

### 3.3 Sequence coverage of RELL1 protein

Sequence coverage is an important parameter in bottom-up proteomics, representing the proportion of the identified peptide sequence length relative to the entire protein sequence length (Shao et al., 2023; Priyanka et al., 2024). Moreover, increased sequence coverage allows for more accurate mapping of post-translational modifications (PTMs), improves the ability to differentiate between protein isoforms, enhances quantitative proteomics, and offers deeper insights into protein structure (Meyer et al., 2011; Shao et al., 2023).

**FIGURE 2:**
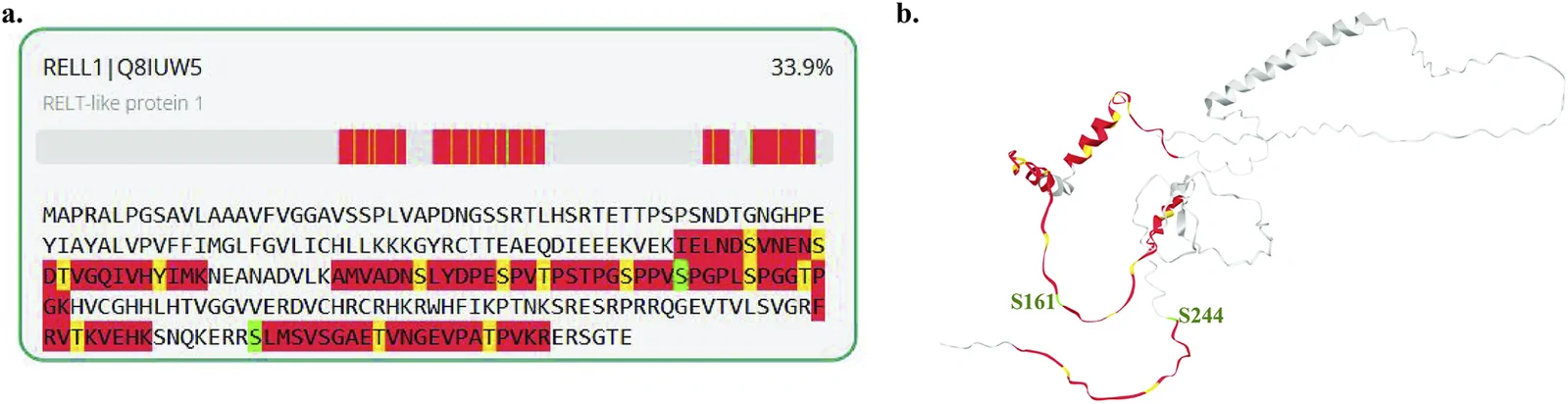
The sequence coverage and the class 1 phosphosites identified from 474 RELL1 phosphoproteomic datasets. **(a)** RELL1 is a 271-amino-acid protein, of which 33.9% of the sequence was covered by the identified phosphopeptides **(b)** The 3D-predicted RELL1 model shows peptides identified in profiling data, with yellow representing the identified RELL1 phosphosites, green representing predominant RELL1phosphosites, and red indicating total sequence coverage.

The analysis of 474 global profile datasets identified 18 peptides corresponding to the RELL1 protein. These peptides covered 33.9% of the full 271-amino-acid sequence of RELL1. This level of sequence coverage highlights the portion of the protein that is potentially detectable by mass spectrometry-based proteomics. The coverage and corresponding structural representation were visualized using the Sequence Coverage Visualizer (SCV) tool and are presented in Figure 2.

### 3.4 The co-regulation network analysis of RELL1 predominant phosphosites and CPOPs

In order to examine the phospho-signaling dynamics associated with RELL1 predominant sites, we carried out a co-regulation analysis of CPOPs. The datasets were initially classified based on the upregulation or downregulation patterns exhibited by CPOPs, corresponding to the expression co-regulation with predominant sites: UUDD (positive co-regulation) and UDDU (negative co-regulation). To determine the likelihood and confidence of expression co-regulation and cross-expression patterns between the phosphosite pairs, a contingency table was created based on a one-sided FET analysis. The number of conditions in which RELL1 predominant phosphosite and their co-expressed protein phosphosites as pairs were extracted into four categories: i) both phosphosite pairs were not identified or reported; one phosphosite was found to be differentially regulated while the other was not; two sites were found to be differentially regulated but exhibited opposite correlation; and two sites were found to be differentially regulated but exhibited positive correlation in expression. To address potential biases and ensure the reliability of our data, we used a confidence criterion that helped to filter out datasets which may have a disproportionate impact on the results due to issues such as a higher number of phosphosites in a few studies or an overabundance of multi-temporal datasets for the same stimulus type. We filtered the protein phosphosites based on the FET P value <0.05; data sets should come in at least three articles and experimental code, and the frequency cutoff of 10 percent.

**FIGURE 3:**
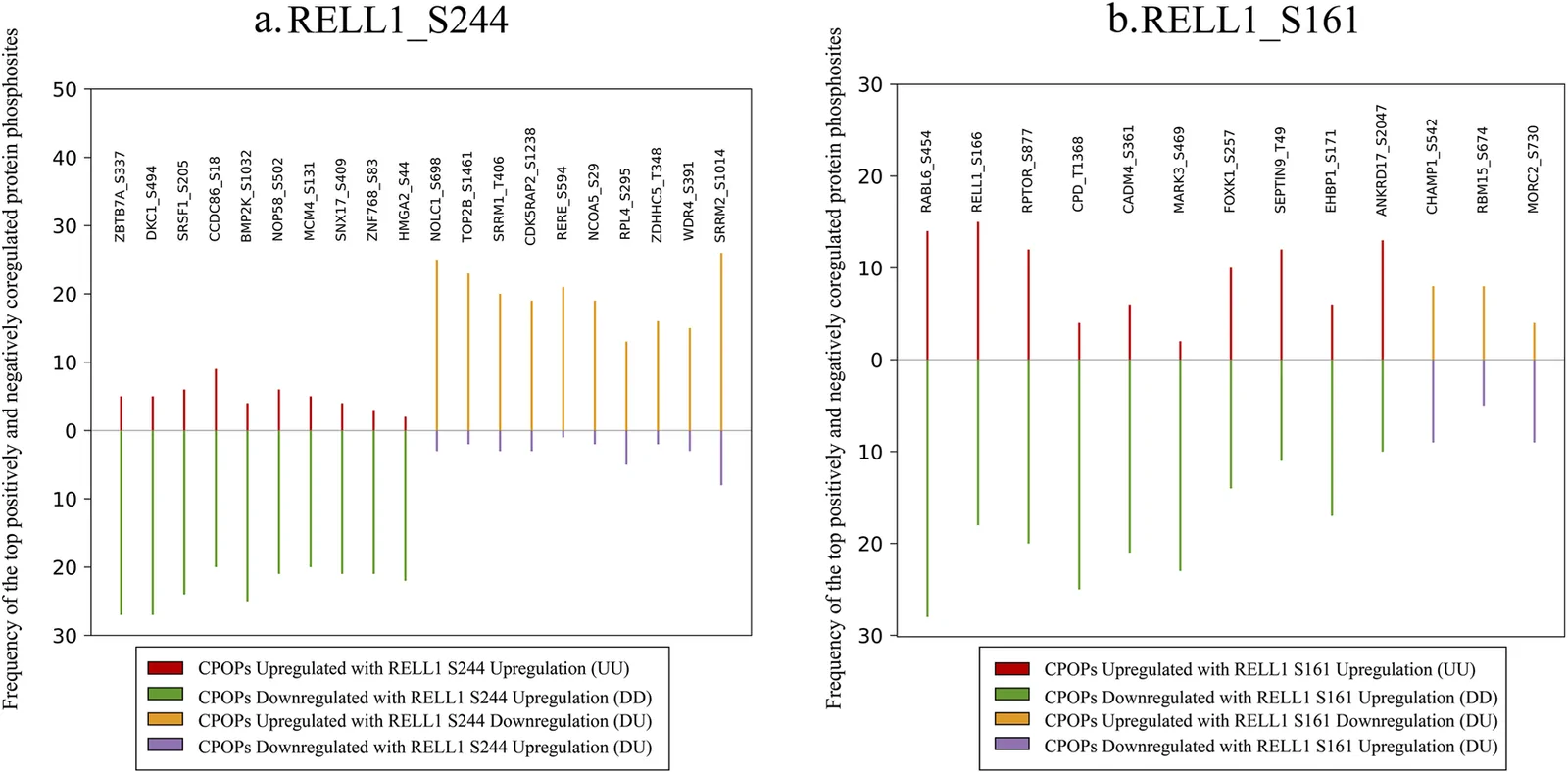
Top high-confidence positively and negatively co-regulated CPOPs of predominant RELL1 phosphosites. **(a)** RELL1_S244 and **(b)** RELL1_S161.

After applying the above thresholds, RELL1 S244 revealed 267 CPOPs that were positively co-regulated and 97 that were negatively co-regulated. Similarly, phosphosite S161 showed 46 positively co-regulated and 3 negatively co-regulated phosphosites within the high-confidence co-regulation criteria and is given in Supplementary Material S2. The top positively and negatively co-regulated proteins are given in Figure 3.

Among the phosphosites positively co-regulated with the S244 site of RELL1, three proteins Zinc Finger and BTB Domain Containing 7A (ZBTB7A)_S337, Dyskerin Pseudouridine Synthase 1 (DKC1)_S494, and Serine And Arginine Rich Splicing Factor 1 (SRSF1)_S205, emerged as top candidates. In addition to positively co-regulated phosphosites, the RELL1 S244 site also exhibited negative co-regulation with Nucleolar and coiled body phosphoprotein 1 (NOLC1)_S698, DNA Topoisomerase II Beta (TOP2B) S1461, and Serine And Arginine Repetitive Matrix 1 (SRRM1)_T406. For the S161 site of RELL1, RAB, Member RAS Oncogene Family Like 6 (RABL6) S454, RELL1_S166, Regulatory Associated Protein Of MTOR Complex 1 (RPTOR)_S877, and Carboxypeptidase D (CPD)_T1368 were the top positively co-regulated CPOPs. Interestingly, RELL1_S166 was identified as an auto-associated site, suggesting potential intramolecular regulation. Among the negatively co-regulated phosphosites, of RELL1_S161, Chromosome Alignment Maintaining Phosphoprotein 1 (CHAMP1) S542, RNA Binding Motif Protein 15 (RBM15)_S674, and MORC Family CW-Type Zinc Finger 2 (MORC2)_S730 were identified.

### 3.5 Co-occurrence pattern analysis of RELL1 phosphosites

**FIGURE 4:**
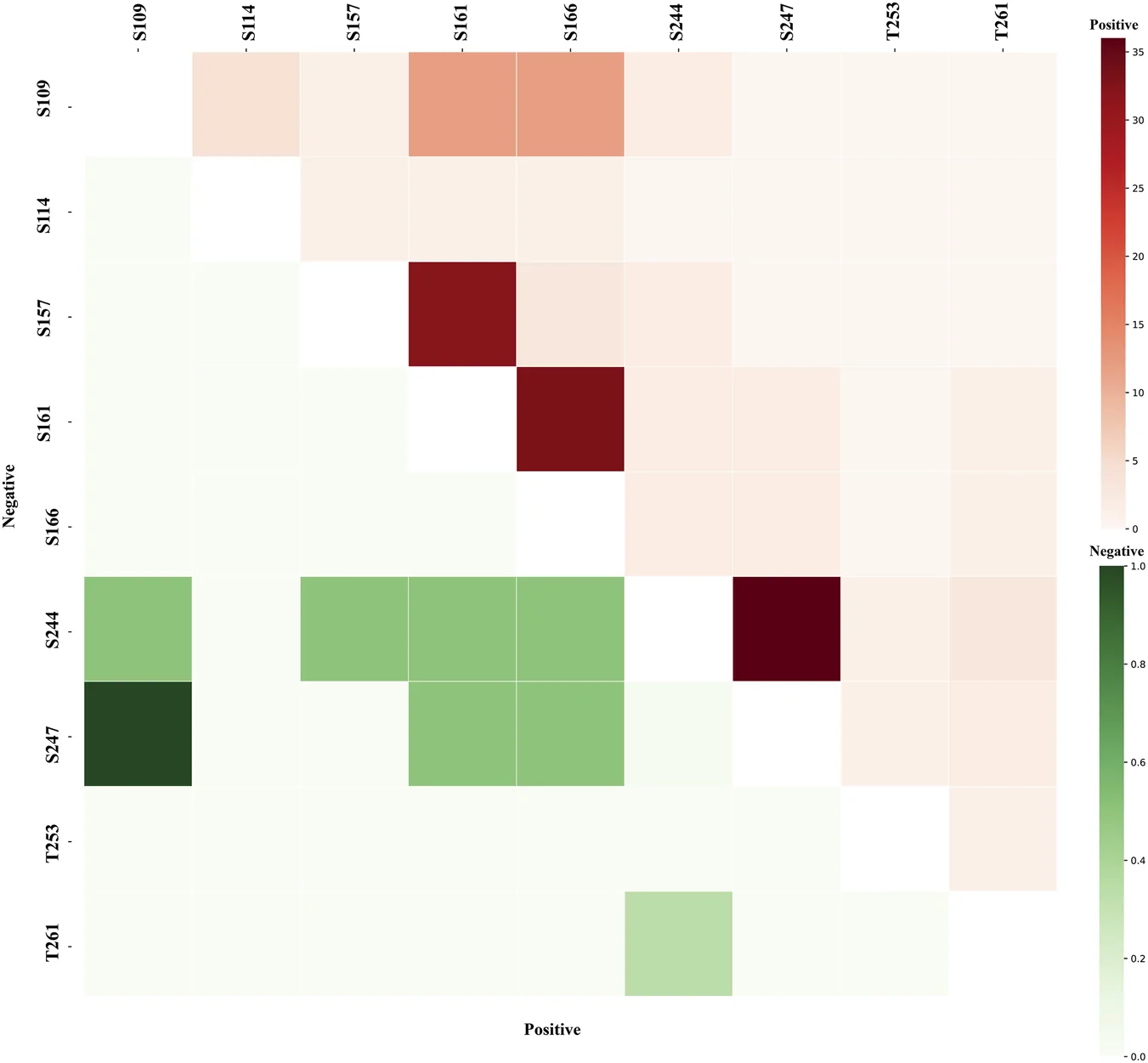
Co-occurrence analysis of RELL1 phosphosites based on expression co-regulation. The heat map represents the co-occurrence pattern of phosphosites, where red indicates positive co-regulation, while green represents negative co-regulation between phosphosite pairs of RELL1, with a color gradient representing the frequency of co-regulation.

Phosphorylation sites that co-occur often tend to exhibit functional similarity and are commonly conserved through evolution (Li et al., 2017). This suggests that such sites together contribute to a particular biological function, localization, or interaction (Sheela et al., 2025; Sekhar et al., 2026). Their conserved nature across species highlights their potential importance in maintaining essential cellular functions, and disruptions at these sites may lead to dysregulation in key signaling pathways. To explore this in the context of RELL1, we analysed the co-occurrence patterns of differential expressions of its phosphosites. Interestingly, the predominant phosphosite S244 exhibited a strong co-occurrence pattern with S247 across 36 differential datasets, while S161 consistently co-occurred with S166 in 33 datasets. This highlights the potential site-level coordination and functional interplay within RELL1’s phosphorylation landscape. The observed co-regulatory relationships between RELL1 phosphorylation sites are visualized in Figure 4, and the co-occurrence data are given in Supplementary Material 3.

### 3.6 Biological insights from co-regulated protein enrichment

Performing enrichment analysis on CPOPs provides broader knowledge of the biological implications of RELL1 phosphorylation. It enables us to identify the potential pathways and cellular activities influenced by its regulation. Such findings suggest that RELL1 can be a part of broader signaling pathways, interconnected networks with outcomes in multiple biological domains. The CPOPs of predominant phosphosites were subjected to conventional gene set enrichment analysis, which was performed using Enrichr (Supplementary Material 4). The major enriched processes were nuclear migration, chromatin organisation, and protein localisation, etc. Interestingly, wound healing, which involves the spreading of epidermal cells, was also seen as enriched.

**FIGURE 5:**
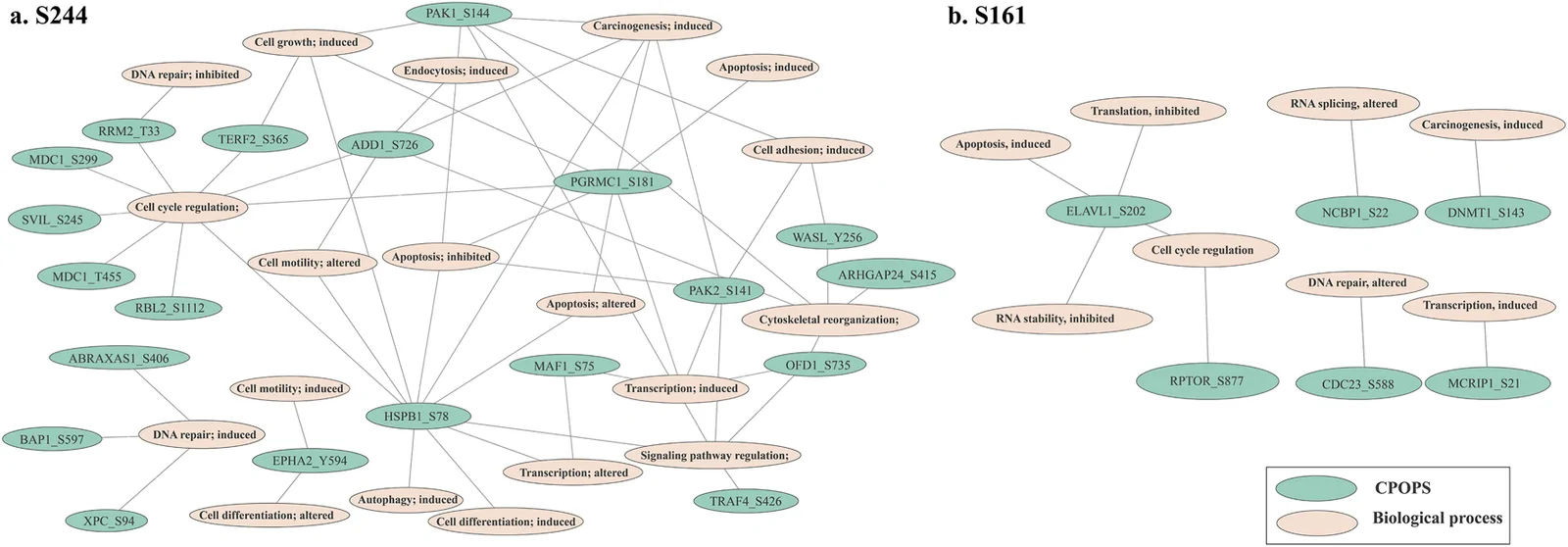
Co-regulation networks of phosphosites functionally associated with RELL1. **(a)** Proteins co-regulated with RELL1_S244 based on shared phosphorylation patterns in our dataset. Associated functions include apoptosis, DNA repair, and cell growth. **(b)** Co-regulated network around RELL1_S161, showing phosphosites associated with processes such as transcription, RNA splicing, and cell cycle regulation.

Analysis of RELL1 at the phosphosite-specific level provided insights into potential phosphoregulatory associations including the activation (“on”) or inhibition (“off”) of specific molecular functions. This enabled us to understand the possible functional roles of individual phosphosites in protein activity and various cellular processes. The identified phosphosites include both activity-induced and activity-inhibited phosphorylation events, as well as sites exhibiting altered activity (altered function without induction or inhibition). Through functional mapping, the co-regulated proteins were found to participate in diverse cellular processes, including regulation of the cell cycle, apoptosis, inhibition of autophagy, transcriptional control, DNA repair mechanisms, modulation of signaling pathways, and reorganization of the cytoskeleton (Figure 5).

Additionally, Enrichr-based disease enrichment analysis of proteins co-differentially regulated with RELL1 phosphorylation revealed enrichment for gene sets associated with neurological and psychiatric disorders. These included neurodegenerative diseases, nervous system dysfunctions, mood-related disorders, Attention deficit/hyperactivity disorder (ADHD) and more. A significant proportion of these proteins showed concurrent upregulation with RELL1 phosphorylation, suggesting that RELL1 phosphoregulation may be associated with molecular pathways linked to these neurological and psychiatric disorders (Supplementary material 4).

### 3.7 Phosphosite-centric analysis of RELL1 predominant sites

#### 3.7.1 Co-differentially regulated kinases and phosphatases associated with RELL1 predominant phosphosites

Cellular signaling modules contain interconnected pathways that regulate a wide range of biological processes. Although the functional importance of signaling cross-talk is well established, the identification and representation of these integrated pathways on a global level is technically challenging. A key mechanism enabling these pathway interactions is protein phosphorylation. This modification is rapid, reversible, and highly specific, allowing the regulation of protein function. Kinases and phosphatases primarily function to regulate post-translational modifications of proteins, which are crucial for controlling cellular signaling pathways (Sacco et al., 2012). To explore the signaling environment of RELL1 phosphorylation, we examined the different phosphosites of kinases and phosphatases that are co-differentially regulated in similar or opposing patterns. The kinases that show positive or negative co-regulation with RELL1 are summarized in Supplementary Material 5A. For the S244 site, positive co-regulation was observed with 16 kinase phosphosites and 1 phosphatase phosphosite. In contrast, the S161 site showed co-regulation with only 3 kinase phosphosites. Only the S244 site had a negative co-regulation with one kinase and phosphatase each.

**FIGURE 6:**
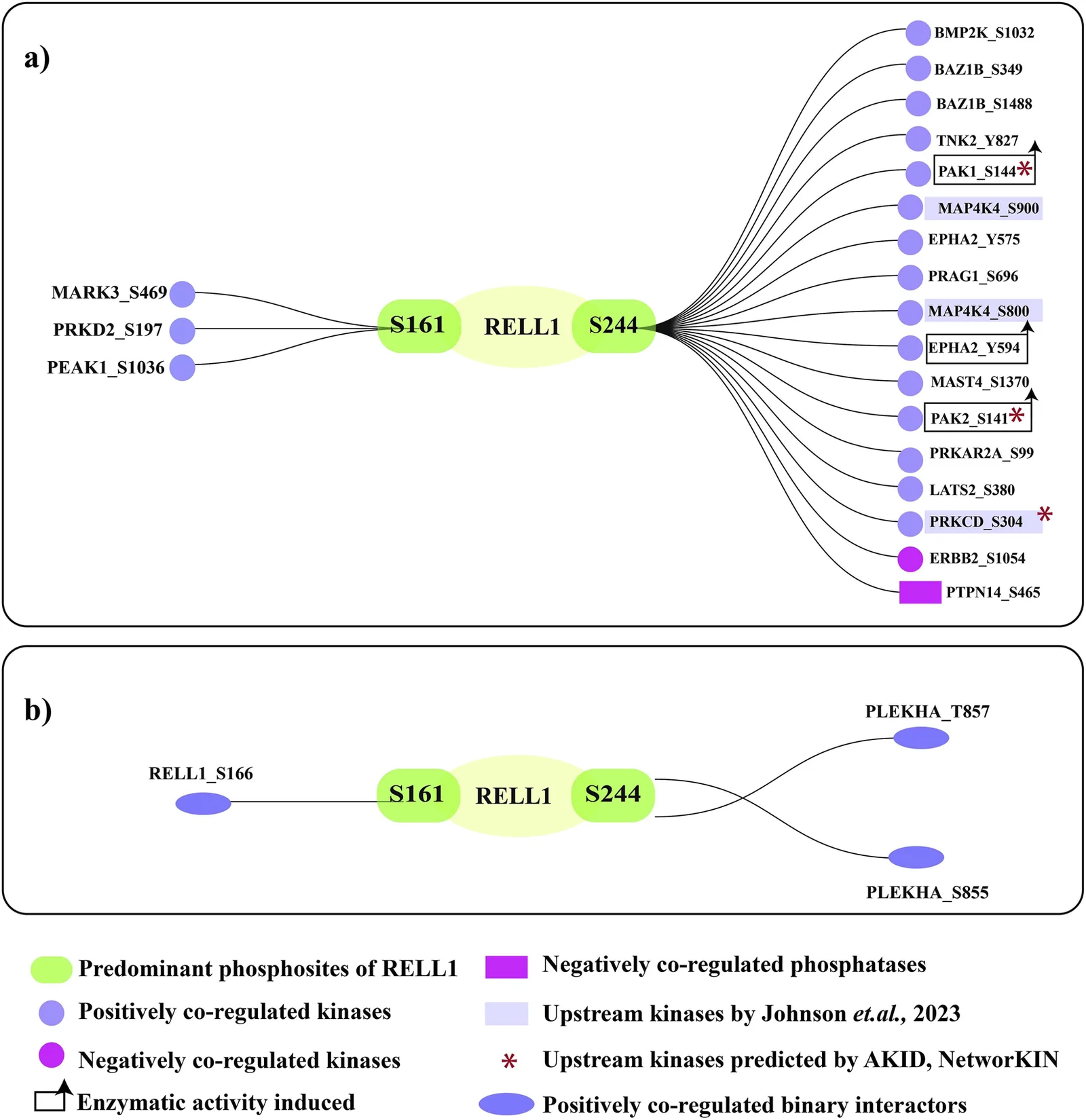
Phosphocentric analysis of RELL1. **(a)** Kinases and phosphatases predicted from RELL1 phosphosite co-regulation analysis **(b)** Binary protein interactors associated with RELL1.

Among the positively co-regulated sites with RELL1 S244, P21 (RAC1) Activated Kinase 1 (PAK1) S144, P21 (RAC1) Activated Kinase 2 (PAK2)_S141, and EPH Receptor A2 (EPHA2)_Y594 are well-characterized activation sites known to induce enzymatic activity, highlighting a potential association of RELL1 in kinase-driven signaling cascades. All the kinases and phosphatase were given in Figure 6a.

#### 3.7.2 Mapping co-regulated phosphosites of predicted upstream kinases of RELL1 predominant sites

Despite the functional relevance of RELL1 in cancer and immune-related processes, the upstream kinases responsible for its phosphorylation remain unidentified. To date, OXSR1 is the only primary kinase identified as an upstream regulator of RELL1. Therefore, further analysis was conducted to identify potential upstream kinases for the RELL1 predominant sites, as predicted by Johnson et al. (2023) using substrate-motif specificity and computational tools such as NetworKIN, AKID, and iKiP-DB.

The S244 site of RELL1 showed positive co-regulation with the S900 and S800 phosphorylation sites of Mitogen-Activated Protein Kinase Kinase Kinase 4 (MAP4K4), as well as the S304 site of Protein Kinase C Delta (PRKCD), as reported by Johnson et al. (2023). Additionally, phosphorylation at S144 of PAK1, S141 of PAK2, and again S304 of PRKCD has been positively co-regulated according to predictive tools such as NetworKIN and AKID, supporting their potential functional linkage in signaling pathways. In contrast, no upstream kinases were identified or predicted for the S161 phosphosite using either the experimentally derived dataset or the computational prediction tools employed in this study.The predicted kinases are highlighted in Figure 6a and are given in Supplementary Material 5B.

#### 3.7.3 RELL1 protein interactome: from direct binders to complex assemblies

The binary interactors and complexes were retrieved from various databases like HPRD, BIND, BioGRID, ConsensusPathDb, CORUM, and RegPhos. The list of binary partners and complex interactors was given in Supplementary Materials 5C,D. The S244 site of RELL1 exhibited positive co-regulation with 54 phosphosites and negative co-regulation with 9 phosphosites, including those within protein complexes. In contrast, the S161 site showed positive co-regulation with 11 phosphosites and no negatively co-regulated sites.

The S244 site of RELL1 showed positive co-regulation with the T857 and S855 sites of Pleckstrin Homology Domain Containing A5 (PLEKHA5), which also appears as a binary interactor of RELL1. For the S161 site of RELL1, the S166 site of RELL1 itself was identified as a positively co-regulated site and also appeared among its binary interactors. The figure showing the binary interactome is given in Figure 6b.

### 3.8 Mapping the phosphosignaling landscape of RELL1

Phosphosignaling of RELL1 is essential for understanding how it integrates into key immune and developmental signaling pathways. Phosphorylation acts as a switch regulating the function, localization, and interaction of RELL1 with adaptor proteins or kinases. It provides insights into the dynamic control of RELL1-mediated responses during cellular stress or inflammation. Mapping its phosphosites helps to identify critical signaling modules that may be disrupted in disease.

To gain a deeper understanding of the functional role of RELL1, a co-regulation analysis was performed on CPOPs that exhibited similar phosphorylation dynamics across relevant conditions. Through this approach, shared signaling modules, upstream kinases, and cellular processes potentially involving RELL1 were identified.

The major known function of RELL1 is the induction of the MAPK/p38 signaling cascade, particularly under conditions of overexpression (Moua et al., 2017). To explore this further, the proteins identified as co-regulated with RELL1 were screened against components of the “MAPK pathway” curated from the Kyoto Encyclopedia of Genes and Genomes (KEGG) database (Kanehisa and Goto, 2000). This comparison revealed several overlapping proteins such as Heat Shock Protein Family B (Small) Member 1 (HSPB1), PAK1, EPHA2, Neurofibromin 1 (NF1), MAP4K4, Jun Proto-Oncogene, AP-1 Transcription Factor Subunit (JUN), and PAK2. This suggests that RELL1 may participate in or influence MAPK signaling networks through shared phosphorylation dynamics. These findings indicate a potential association between RELL1 in MAPK/p38-mediated cellular processes. Another established function of RELL1 is the induction of apoptosis when overexpressed (Moua et al., 2017). This pro-apoptotic role was further supported by our enrichment analysis, where several co-regulated proteins were found to be involved in apoptotic pathways. These observations suggest that RELL1 may contribute to cell death signaling not only through direct overexpression but also via coordinated phosphoregulatory events within broader apoptotic networks.

Additionally, the molecular functions exhibited by the CPOPs of the predominant sites include cell cycle regulation, autophagy inhibition, apoptosis induction, DNA repair, cytoskeletal reorganization, signaling pathway regulation, and other related functions.

From KEGG-based screening of autophagy-related genes, PRKCD and Baculoviral IAP Repeat Containing 6 (BIRC6) are associated with RELL1 S244, as well as RPTOR and AKT1 Substrate 1 (AKT1S1) are associated with RELL1 S161, suggesting that RELL1 may be functionally linked to autophagy regulation through distinct phosphosignaling routes.

**FIGURE 7:**
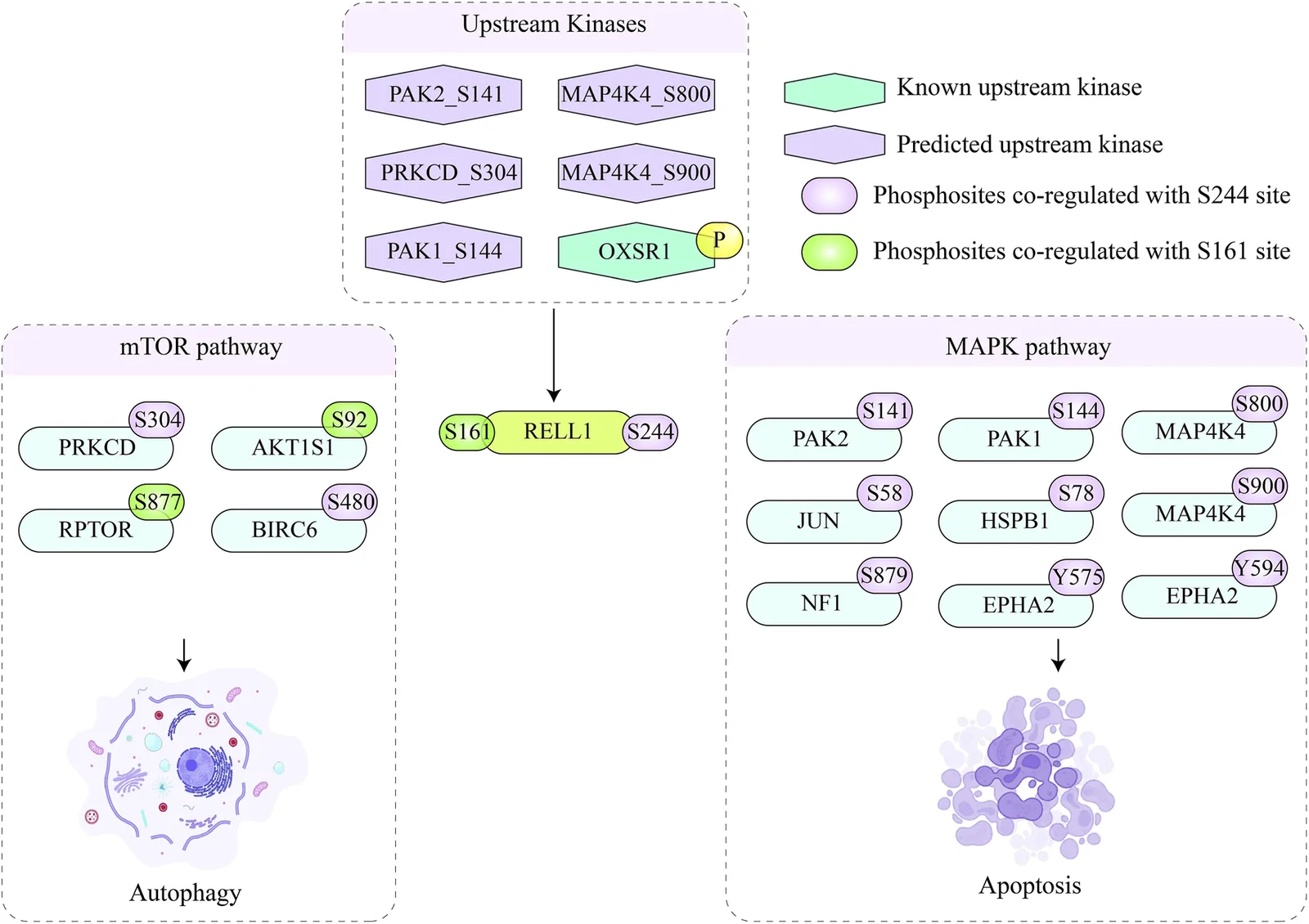
Phosphosignaling network of RELL1. RELL1 phosphosites (S161, S244) along with its known kinase OXSR1 and the predicted upstream kinases are shown with associated proteins mapped to MAPK and mTOR pathways. These interactions connect RELL1 to apoptosis and autophagy.

Additionally, PLEKHA5, a binary interactor identified for RELL1, plays a critical role in brain development through its brain-specific splice variant L-PLEKHA5, which becomes dominant in the later stages of embryonic development (Yamada et al., 2012). Importantly, PLEKHA5 also promotes brain metastasis in melanoma and is a biomarker and mediator of melanoma brain metastasis (Jilaveanu et al., 2015). Since RELL1 has been implicated in glioblastoma as well, its interaction with PLEKHA5 strengthens the hypothesis that this axis could be functionally significant in neurodevelopment and brain tumorigenesis, especially in glioblastoma. An overview of these findings is represented in Figure 7.

## 4 Discussion

RELL1 remains a largely unexplored transmembrane protein, with limited understanding of its biological function and regulatory mechanisms. Although emerging studies suggest that RELL1 is involved in cancer, immune responses, and inflammation, the specific molecular pathways regulated by RELL1 remain unexplored (Cusick et al., 2023). The signaling pathways, functional roles, and interaction networks of a protein are regulated by its PTMs. Among the reported PTMs of RELL1, phosphorylation remains the most prominent modification observed across high-throughput proteomic datasets. Based on *in vitro* kinase assay, OXSR1 and STK39 were shown to phosphorylate RELL1 (although its target phosphosites in RELL1 were not determined) by Cusick et al. (2006). Despite its prevalence, the phosphorylation landscape of RELL1 remains poorly defined, with no prior studies offering a systematic view of its phosphosites or their regulatory mechanisms. This gap is particularly significant considering the central role played by phosphorylation in modulating signaling pathways and dynamic cellular responses. In this study, we undertook a comprehensive phosphoproteomic approach to investigate RELL1 signaling, aiming to identify key phosphorylation sites, upstream regulators, co-regulated signaling networks, and associated disease pathways.

We analysed 150 differential datasets and 474 profile datasets containing class-1 RELL1 phosphosites to explore the phosphoregulatory network of RELL1. S244 and S161 were identified as the predominant sites based on their recurrent detection in multiple datasets, and subsequent co-regulation analysis of CPOPs was performed. Highly co-regulated phosphosites, those that show similar expression or activation patterns across multiple conditions, are usually a result of functional dependency (Ayati et al., 2022). These co-regulation trends suggest that such phosphosites can be the target of a single kinase or share some signaling cascades. As a result, they are likely to contribute to coordinated regulation of key cellular events such as cell cycle progression, apoptosis, stress responses, or differentiation. Their coordinated activity under dynamic conditions and stimuli indicates that they may perform an integrated function rather than an independent function. Therefore, understanding these co-regulated patterns can provide valuable insights into the shared regulatory pathways, uncover novel signaling networks, and even suggest potential therapeutic targets in disease contexts where such pathways are dysregulated.

The co-regulation analysis revealed 267 CPOPs that were positively co-regulated and 97 that were negatively co-regulated for the S244 site. The S161 site showed 46 positively co-regulated and 3 negatively co-regulated phosphosites. These top positively co-regulated proteins at the S244 site are known to be involved in transcriptional control, ribonucleoprotein complex assembly, and mRNA splicing, respectively. Among these, ZBTB7A acts as a transcription factor involved in immune regulation and cancer progression (Zhu et al., 2018), DKC1 is a nucleolar protein critical for ribosomal RNA modification and telomerase RNA stability, and its dysfunction has been implicated in X-linked dyskeratosis congenita, bone marrow failure, and various cancers (Parry et al., 2011). SRSF3 regulates mRNA metabolism and various biological processes, with potential as a therapeutic target in cancer, aging, neurological, and cardiac disorders (More and Kumar, 2020). Likewise, the negatively co-regulated phosphosites NOLC1 is a nucleolar phosphoprotein involved in rRNA synthesis, DNA replication, and transcriptional regulation (Zhai et al., 2023); TOP2B regulates DNA topology during transcription and chromatin remodeling (Vávrová and Šimůnek, 2012); and SRRM1, a spliceosome-associated factor, is implicated in alternative splicing regulation and localization to nuclear speckles (Song and Ma, 2022). The negative co-regulation of these phosphosites with RELL1 suggests potential antagonistic or divergent regulation in nuclear processes such as RNA splicing, transcription, and ribosome biogenesis.

The positive co-regulation of S161 includes RABL6, which is implicated in cell cycle control and tumor suppression, while RPTOR is a critical component of the mTORC1 complex, linking RELL1 signaling to metabolic and growth pathways (Zhang et al., 2013; Stelzer et al., 2016). CPD enriched in the trans-Golgi network is involved in the processing and trafficking of secretory proteins, with its localization regulated by casein kinase 2-dependent phosphorylation (Kalinina et al., 2002). CPD is found to be co-regulated by both sites. Furthermore, the negatively co-regulated phosphosites, CHAMP1 is a mitotic regulator associated with chromatin structure and genome stability through its interaction with Chromodomain Y Like 2 (CDYL2) and Pogo Transposable Element Derived With ZNF Domain (POGZ) (Siouda et al., 2023). RBM15 plays a role in myelopoiesis and inhibits myeloid differentiation in hematopoietic cells by stimulating Notch signaling through Recombination Signal Binding Protein For Immunoglobulin Kappa J Region (RBPJ) (Ma et al., 2007). MORC2 promotes cancer stemness and tumorigenesis by facilitating DNA methylation-dependent silencing of Hippo signaling, making it a potential target for cancer therapeutics (Wang et al., 2018). The negative co-regulation of these sites may reflect opposing roles in transcriptional regulation, chromatin organization, and cell cycle dynamics relative to RELL1 S161 signaling. Interestingly, TRAF4_S426 was identified among the CPOPs. TRAF4 represents the only TRAF member that negatively regulates NF-κB signaling (Marinis et al., 2012).

In previous studies, RELL1 induced apoptosis and activated the p38 MAPK signaling cascade, functioning through a mechanism distinct from classical TNFR1-mediated cell death pathways (Moua et al., 2017). However, the precise molecular intermediates and regulatory phosphosites involved in this process remain undefined. So, for a better understanding of the functional role and regulatory mechanisms of RELL1, an analysis of CPOPs was performed. Interestingly, several proteins, such as HSPB1, PAK1, EPHA2, NF1, MAP4K4, JUN, and PAK2, co-regulated at the S244 predominant phosphosite of RELL1, were identified as components of the MAPK signaling pathway based on KEGG pathway analysis. Among these, PAK1, PAK2, and MAP4K4 were not only part of the MAPK signaling pathway but were also identified as predicted upstream kinases of RELL1 at the S244 site. PRKCD is another predicted upstream kinase at the S244 site that can also activate the MAPK pathway, specifically the ERK1/2 and JNK pathways, through different mechanisms (Ueda et al., 1996; Raghu et al., 2025). Together, this suggests a strong functional association between RELL1 and MAPK-mediated signaling regulation. MAPK are typically activated in response to stress and are well known for inducing apoptosis. This duality allows cells to fine-tune apoptotic responses based on physiological context (Yue and López, 2020). Kinases such as PAK1, PAK2, MAP4K4, and PRKCD, which are involved in MAPK pathways, function as upstream regulators influencing whether MAPK signaling drives pro-survival or pro-death outcomes. Their strong co-regulation with RELL1 in our study suggests a potential intersection where RELL1 may have a role in apoptosis through MAPK-dependent mechanisms. Interestingly, no candidate upstream kinase was identified for the RELL1 S161 phosphosite using either experimentally derived kinase-substrate data or computational prediction tools. This finding does not necessarily indicate that S161 is not regulated by phosphorylation. Rather, it may reflect limitations of current kinase prediction resources, context-dependent phosphorylation events, or regulation by atypical or yet-to-be-characterized kinases. Therefore, further experimental studies are required to identify the upstream kinases responsible for S161 phosphorylation and to determine its biological significance.

RELL1 has been shown to inhibit autophagy by enhancing mTOR activity, thereby suppressing autophagic clearance in macrophages during *Mycobacterium tuberculosis* infection (Feng et al., 2020). Supporting this, several autophagy-associated genes identified from the KEGG pathway, PRKCD and BIRC6 (linked to the S244 site) and RPTOR and AKT1S1 (linked to the S161 site) were found to be co-regulated with RELL1 phosphosites. These findings collectively suggest that RELL1 may influence autophagy by acting on the mTOR signaling axis, further reinforcing its potential role in stress and immune-regulated cellular pathways.

Although RELL1 is primarily localized at the plasma membrane, the enrichment analysis of its co-regulated phosphoproteins revealed significant enrichment in nuclear and intracellular processes, including nuclear migration, RNA processing, chromatin organization, and regulation of gene expression. Additionally, processes such as cell cycle transition (G1/S), apoptosis, and cytoskeleton organization were also enriched, suggesting that RELL1 may initiate signaling events at the membrane that propagate into the nucleus or cytoplasm. RELL1 protein has been shown to activate NF-κB and AP-1 in macrophages during *Mycobacterium tuberculosis* infection, further supporting its pro-inflammatory role (Feng et al., 2020). Supporting this, our enrichment analysis showed involvement in wound healing and inflammatory signaling, suggesting RELL1 may have a potential role in immune activation and tissue repair.

RELL1 is evolutionarily conserved across multiple species, and interestingly, our analysis revealed that the two predominant phosphorylation sites, S244 and S161, are also conserved. This suggests that these sites may hold important regulatory functions that have been maintained through evolution, further supporting their potential biological significance. Evidence from knockout mouse models suggests that RELL1 plays a critical role in nervous system function (Groza et al., 2023). The phenotypic analysis of RELL1 knockout mice, generated by the deletion of exon 4 in a C57BL/6NJ mice, exhibited significant abnormalities in neurological function, highlighting its importance in brain physiology (Dickinson et al., 2016). Data from the International Mouse Phenotyping Consortium revealed that deletion of exon 4 in the RELL1 gene, leading to a truncated protein, caused critical neurological and behavioral abnormalities (Cusick et al., 2023; Groza et al., 2023).

Disease enrichment analysis using the Jensen Disease database revealed that several conditions were significantly associated with RELL1-related co-regulated phosphoproteins. Notably, many of the top-enriched categories were neurological and neurodevelopmental disorders, including nervous system disease, brain disease, central and peripheral nervous system diseases, neuropathy, intellectual disability, cognitive disorder, dementia, attention deficit hyperactivity disorder (ADHD), and spinocerebellar ataxia with axonal neuropathy 2 (SCAN2). In addition, sensorineural hearing loss and inner ear disease were also enriched, aligning with phenotypic findings from RELL1-deficient mouse models (Grissa et al., 2022). Beyond neural disorders, enrichment was also observed for cancer-related categories (e.g., adenocarcinoma, bile duct cancer, testicular cancer, organ system cancers), as well as lipodystrophy, connective tissue disease, and mood disorders such as major depressive disorder and melancholic depression. These findings suggest that RELL1 may be associated with signaling pathways linked to neurological and neurodevelopmental disorders. However, these associations are based on computational methods for enriching co-regulated proteins and should be interpreted cautiously until experimentally validated.

In summary, this study provides the first comprehensive phosphoproteomic analysis of RELL1, uncovering its potential regulatory roles through key phosphorylation sites S244 and S161. The integration of co-regulated phosphosite networks, kinase predictions, functional enrichment, and disease associations suggests that RELL1 may have potential associations in intracellular processes such as apoptosis, autophagy, chromatin remodeling, and cytoskeletal organization. Although RELL1 is known to function as an adaptor protein within the RELT family and has been reported to interact with RELT to mediate downstream signaling. However, phosphoproteomic evidence for RELT remains highly limited, as also reflected in PhosphoSitePlus, where only a small number of phosphosites have been reported (Hornbeck et al., 2012). Although RELT phosphosites were detected in the initial curated datasets, they did not satisfy the predefined high-confidence filtering criteria and were therefore not included in the final analysis. This highlights an important gap in the current understanding of RELT family phosphoregulation, and future studies integrating phosphosite-specific analyses of both RELT and RELL1 will be valuable for understanding the coordinated regulation of the RELT-RELL1 signaling axis and its biological significance.

Furthermore, enrichment for neurological, cognitive, and inflammatory disorders, alongside supporting mouse phenotype data, points toward a previously unrecognized role for RELL1 in neurodevelopmental and neurodegenerative disease contexts. Together, these findings establish a foundational framework for future investigations into the molecular mechanisms of RELL1 and its potential as a therapeutic target in disease regulation.

## 5 Limitations and future directions

The present study provides a comprehensive phosphosite-centric analysis of RELL1 using large-scale publicly available phosphoproteomic datasets. However, several limitations should be considered when interpreting the findings. First, the analyses are entirely computational and hypothesis-generating; therefore, the identified phosphoregulatory associations, predicted upstream kinases, and functional implications require experimental validation. Second, the curated datasets were generated using diverse experimental conditions, mass spectrometry platforms, phosphopeptide enrichment strategies, and data processing workflows. Since uniformly processed raw data were not available for all studies, cross-dataset normalization and batch-effect correction could not be performed. Although stringent filtering criteria were applied to minimize technical variability and improve the robustness of the analysis, residual heterogeneity across datasets cannot be completely excluded.

Furthermore, phosphosite co-regulation represents statistical association rather than direct regulatory or causal relationships. Likewise, the predicted kinase-substrate interactions should be considered candidate associations until confirmed experimentally. The absence of predicted upstream kinases for the RELL1 S161 phosphosite may reflect limitations of the currently available phosphoproteomic datasets and prediction resources, context-dependent phosphorylation, or regulation by yet unidentified kinases. Future studies should focus on experimentally validating the predominant phosphosites and their predicted upstream kinases using biochemical and cellular approaches, including phosphosite-directed mutagenesis, kinase assays, and functional studies.

## 6 Conclusion

Our study presents the first phosphoproteomic analysis of poorly characterized RELL1, revealing its possible regulatory and functional roles. The in-depth global phosphoproteomic dataset analysis of RELL1 led to the identification of two predominant sites, S244 and S161 respectively, based on the detection frequency. Co-regulated phosphosite mapping, identification of predicted upstream kinases, and protein interactome revealed the role of RELL1 in cell cycle regulation, nuclear migration, apoptosis, MAPK signaling, etc. Furthermore, the enrichment analysis suggests a potential link between RELL1 and neurological disorders, indicating that it may have broader physiological and pathological relevance than previously appreciated. Our findings provide a strong foundation for understanding the regulatory role of RELL1 in cellular signaling, and also open avenues for further targeted studies. It is important to acknowledge that the current findings are data-driven, and the observed patterns and site interdependencies will serve as a valuable basis for future hypothesis-driven research.

## Data Availability

The original contributions presented in the study are included in the article/Supplementary Material, further inquiries can be directed to the corresponding authors.
